# Do early-life circumstances predict late-life suicidal ideation? Evidence from SHARE data using machine learning

**DOI:** 10.3389/fpsyt.2024.1426876

**Published:** 2024-11-14

**Authors:** Xu Zong, Huaiyue Wang

**Affiliations:** ^1^ Helsinki Institute for Demography and Population Health, Faculty of Social Sciences, University of Helsinki, Helsinki, Finland; ^2^ Max Planck - University of Helsinki Center for Social Inequalities in Population Health, Helsinki, Finland; ^3^ School of Public Administration, Faculty of Economics and Management, East China Normal University, Shanghai, China

**Keywords:** childhood adversity, suicide in older adults, mental health of older adults, XGBoost, artificial intelligence, life course, health aging, social determinants of health

## Abstract

**Background:**

A number of studies have demonstrated that suicidal ideation in late life is associated with early-life circumstances. However, the importance of early-life circumstances in predicting suicidal ideation is not entirely clear. This study aims to use a machine learning approach to evaluate the importance of 32 early-life circumstances from six domains in predicting suicidal ideation in old age.

**Methods:**

The data in this study come from a cross-national longitudinal survey, the Survey of Health, Aging and Retirement in Europe (SHARE). Participants recalled information on early-life circumstances in SHARE wave 7 and reported suicidal ideation in SHARE wave 8. The XGBoost model was employed to evaluate the importance of 32 circumstances in six domains (early-life socioeconomic status, early-life health and healthcare, early-life relationship, etc.) in predicting the suicidal ideation of middle-aged and older adults over 50.

**Results:**

There were 46,498 participants in this study, of which 26,672 (57.36%) were females and 19,826 (42.64%) were males. XGBoost showed a strong predictive performance, with an area under the curve of 0.80 and accuracy of 0.77. Top predictors were mainly in the domains of childhood relationship, childhood socioeconomic status, childhood health, and healthcare. In particular, having a group of friends most critically influences suicidal ideation in old age.

**Discussion:**

These findings suggest that early-life circumstances may modestly predict suicidal ideation in late life. Preventive measures can be taken to lower the risk of suicidal ideation in middle-aged and older individuals.

## 1 Introduction

With the aging population increasing all over the world, the suicide of older adults has become a crucial public health problem (1). Large empirical studies have indicated that suicidal ideation is closely associated with completed suicide (2). In Europe, the lifetime prevalence of suicidal ideation was 7.8%, and there are significant variations in suicidal ideation by sex among middle-aged and older adults (3, 4). There is a need for knowledge about the causes and prevention of suicidal ideation in older adults. However, most studies exploring the predictors have mainly focused on late-life predictors (5, 6). Previous studies have established strong associations between (7, 8) childhood adversity and the increased likelihood of suicide in late life, including factors like childhood physical health (7–10), psychological, physical, and sexual abuse (7, 8, 10–13), family socioeconomic status (7, 9), parental divorce (10), exposure to holocaust and wars (14, 15), and hunger (16). In particular, childhood physical health, including physical illness and self-reported health, is closely associated with the risk of suicidal ideation in old age (9, 10). For instance, a US study revealed that individuals with poor health in childhood had 1.99 times greater odds of suicidal ideation than healthier counterparts (9). The correlation may stem from the fact that poor health during childhood increases the risk of mental health problems, chronic diseases, and poor social support (17–19), all of which can contribute to suicidal ideation in old age. Although the relationship between childhood adversity and suicidal ideation is widely established, the importance of these early-life circumstances in elderly suicide has not been sufficiently explored. Additionally, previous studies have demonstrated that there are variations in suicidal ideation by sex (20–22), pointing out the gender inequality in this phenomenon. The existing literature investigated the associations between early-life circumstances and suicidal ideation in late life mainly using conventional statistical models that struggle to deal with non-linear relationships and high-dimensional problems. Machine learning models show promising potential in coping with such problems and have been widely used in studies of medicine and psychology (23–25). So far, machine learning models have been applied to predict suicide using several datasets such as healthcare data, clinical data, registry data, and national survey data (26–29). For example, a study of the use of machine learning to predict the suicidal ideation of Korean adults, showed that the predictive performance of machine learning models outperformed conventional statistical models such as the logistic regression model (30). However, these studies applied machine learning models to predict suicidal ideation risk using late-life factors rather than early-life factors, which may make it difficult to comprehensively understand the relative importance of life-course factors in predicting elderly suicidal ideation (31, 32). In addition, these studies mostly utilized single-county datasets.

In this study, we aimed to leverage XGBoost, a machine learning model, and cross-national survey data, to examine the importance of early-life circumstances in predicting suicidal ideation in late life. In addition, 32 early-life predictors were constructed based on the rich information about life history from the Survey of Health, Aging and Retirement in Europe (SHARE). The survey collected various information about individuals through face-to-face computer-aided personal interviews. A range of studies have used SHARE data to identify the risks of suicidal ideation in older adults (33–35), but few of them examined the early-life risks. Strengthening the understanding of the life-course predictions of suicidal ideation can enhance targeted prevention measures for older populations who have experienced adverse early-life circumstances.

## 2 Data and methods

### 2.1 Data

#### 2.1.1 Participants

The data used in this study come from SHARE, a longitudinal cross-national survey that has been running from 2004 to the present (36). Until 2024, it has covered 28 European countries and Israel, and provided rich information on middle-aged and aged adults in health, social, economic, and other aspects. This study obtained the data of participants who were interviewed in SHARE wave 7 and wave 8 and were aged 50 years or above. After excluding the participants who were aged below 50 years, the final analytical samples consisted of 46,498 participants.

#### 2.1.2 Assessment of suicidal ideation

In SHARE wave 8, participants were asked ‘In the last month, have you felt that you would rather be dead?’ This study constructed a binary variable to assess suicidal ideation. The answer ‘Any mention of suicidal feelings or wish to be dead’ is assigned a value of 1, and the answer ‘No such feelings’ is assigned a value of 0. Previous studies offered evidence for a binary measurement of suicidal ideation of older adults (33–35).

#### 2.1.3 Assessment of predictors

Participants were asked to recall their life history in SHARE wave 7. Derived from SHARE wave 7, 32 early-life circumstances were constructed, including six domains: childhood socioeconomic status, childhood health and healthcare, childhood relationship, childhood war, childhood residence conditions, and childhood cognition. Previous studies have shown that these predictors contribute to the occurrence of suicidal ideation in late life (8, 11, 15, 37). Moreover, we included 23 late-life predictors that have been proven to influence suicidal ideation in old age, such as gender, marital status, age, education level, health conditions, and economic situation (5, 38, 39). Detailed descriptions of these predictors can be found in **Appendix A**, **B**. Considering the potential high correlation between the predictors, we calculated the correlation matrix for all predictors, which is shown as a heatmap in **Appendix D**. Additionally, it was observed that the majority of the predictors are not highly correlated.

### 2.2 Methods

XGBoost, which stands for eXtreme Gradient Boosting, is a powerful machine learning model with exceptional performance and efficiency in classification and regression. This model is based on the gradient boosting framework and good at dealing with large datasets and complex tasks. It has recently been applied to examine the predictors of suicide (40–43).

**Figure 1:**
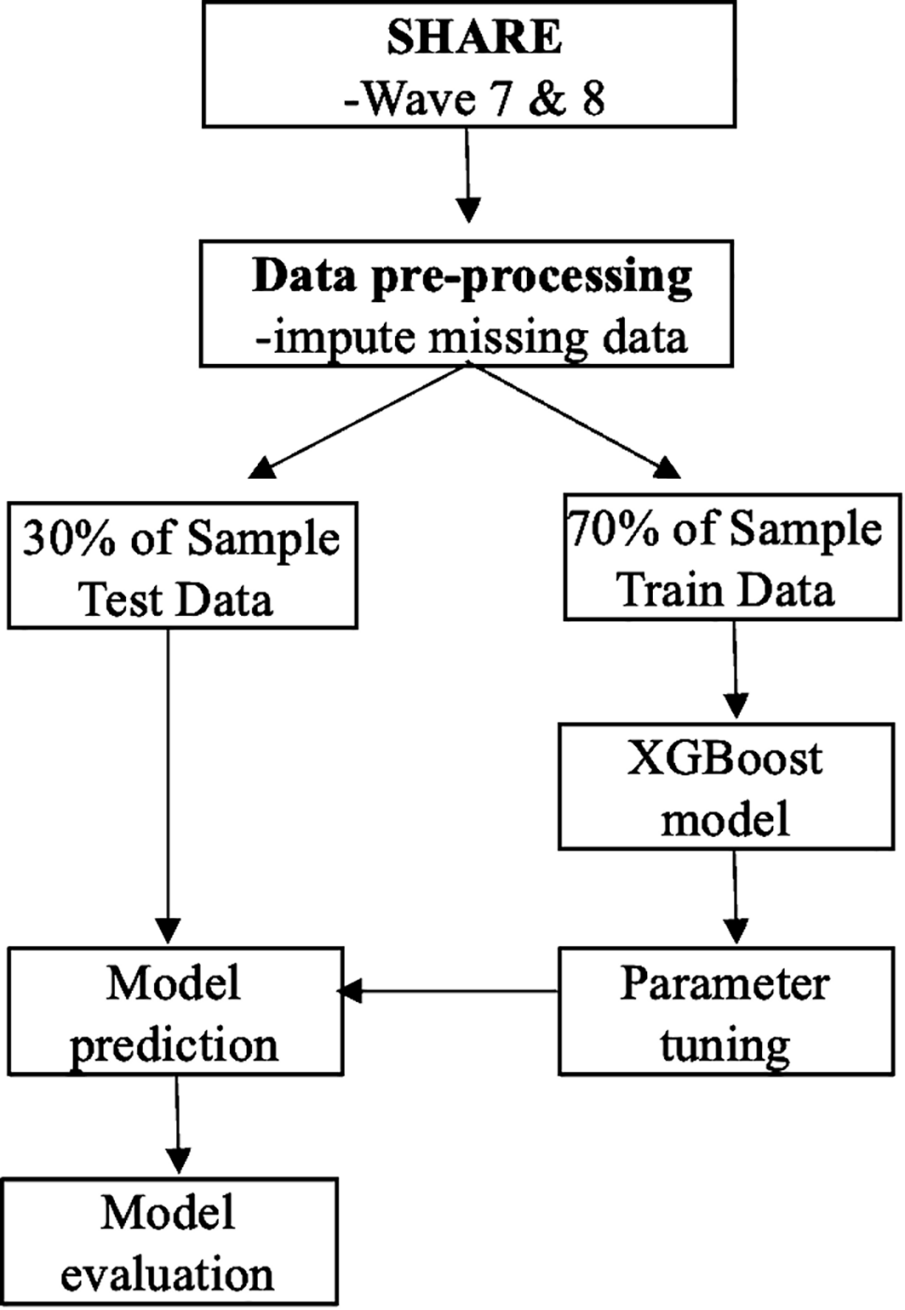
Methodology process.

In this study, XGBoost was applied to evaluate the importance of 32 early-life circumstances in predicting suicidal ideation in old age. This model has several unique advantages in prediction compared with conventional linear regression models. First, XGBoost is good at capturing non-linear relationships between predictors and outcomes, which is often missed by linear models. Second, XGBoost can deal with multicollinearity effectively in which features are highly correlated. Third, XGBoost is a non-parametric model and allows the capture of a wider variety of patterns in the data. XGBoost Python package was used to develop the XGBoost model in this study. The ‘xgboost’ library in Python (version 3.10.9) was used to develop the XGBoost model in this study. **Figure 1** shows the methodology process, including data pre-processing, model development, and model evaluation.

#### 2.2.1 Data pre-processing

In this study, the average percentage of missing data across all variables was approximately 11%. Random forest imputation takes into account non-linearities and interactions and does not require the specification of a specific regression model (44). We employed random forest imputation to minimize bias from missing data, ensuring that our analysis remained robust and accurate.

#### 2.2.2 Model development

To evaluate the generalization ability of the XGBoost model, the data of this study was first divided into training data and test data, comprising 70% and 30% of the data, respectively. Parameter tuning was performed exclusively on the training data using 10-fold stratified cross-validation with GridSearchCV, which ensured the balance of the outcome variable across folds. In addition, scale_pos_weight of the XGBoost model was adjusted to further accommodate the class imbalance problem as only a few older adults (5.88%) had suicidal ideation. After identifying the optimal hyperparameters, the model was re-trained on the whole training data. Finally, we evaluated the model’s predictive performance on the test data.

#### 2.2.3 Model evaluation

We used a range of indicators to evaluate the predictive performance of the XGBoost model. These indicators include the area under the curve (AUC), accuracy, positive predictive value (PPV), negative predictive value (NPV), sensitivity, specificity, and F1 score.

#### 2.2.4 Feature importance

In this study, the XGBoost model provides the importance of each feature by calculating each feature’s importance score. This is based on the ‘gain’ method.

## 3 Results

### 3.1 Descriptive analysis

In this study, the total number of final analytic samples was 46,498. Of these participants, 2,736 (5.88%) individuals aged 50+ years reported having suicidal ideation in the last month, whereas 43,762 (94.12%) did not. To assess whether there were statistically significant differences in the bivariate relationships between having suicidal ideation and not having suicidal ideation with other variables in the study, we conducted a *t*-test for continuous variables and a chi-squared test for categorical variables. The mean age of participants with suicidal ideation was 73.75 years (SD = 10.25), which was higher than those without at 70.19 years (SD = 9.23). Females (7.09%) exhibited higher rates of ideation than males (4.27%), and the prevalence was notably higher among widowed (10.71%), divorced (7.86%), and separated (7.97%) individuals than among married ones (4.24%). Education also played an important role, with those having less than an upper secondary education showing the highest ideation rate (8.38%) and those with tertiary education showing the lowest rate (3.75%). Although differences between urban (5.81%) and rural (6.03%) participants were not significant, living in nursing homes was associated with a significantly higher rate of suicidal ideation (14.73%). Across countries, the prevalence ranged from 3.35% in Northern Europe to 6.86% in Western Europe. Overall, participants who are female, unmarried, widowed, or divorced, have lower levels of education, and live in nursing homes and Western European countries, are more susceptible to harboring suicidal ideation. Detailed characteristics of the sample refer to **Table 1**.

**Table 1:** The demographic characteristics of participants in this study.

Characteristics	Having suicidal ideation		Not having suicidal ideation		Total		*p*-value
	n	%	n	%	n	%	
Total	2,736	5.88	43,762	94.12	46,498	100	
**gender**							<0.001
Male	846	1.82	18,980	40.82	19,826	42.64	
Female	1,890	4.06	24,782	53.30	26,672	57.36	
**marital_status**							<0.001
Married	1,305	2.81	29,451	63.34	30,756	66.14	
Registered partnership	32	0.07	591	1.27	623	1.34	
Separated	38	0.08	439	0.94	477	1.03	
Divorced	300	0.65	3,515	7.56	3,815	8.20	
Widowed	914	1.97	7,618	16.38	8,532	18.35	
Never married	147	0.32	2,148	4.62	2,295	4.94	
**education**							<0.001
Less than upper secondary education	1,360	2.92	14,868	31.98	16,228	34.90	
Upper secondary and vocational training	970	2.09	18,483	39.75	19,453	41.84	
Tertiary education	406	0.87	10,411	22.39	10,871	23.26	
**rural_urban**							> 0.1
Urban	1,819	3.91	29,482	63.40	31,301	67.32	
Rural	917	1.97	14,280	30.71	15,181	32.65	
**living_in_nursing home**							<0.001
No	2,660	5.72	43,322	93.17	45,982	98.89	
Yes	76	0.16	440	0.95	516	1.11	
**region**							<0.001
Northern Europe	190	0.41	5,484	11.79	5,674	12.20	
Southern Europe	512	1.10	8,103	17.43	8,615	18.53	
Eastern Europe	1,033	2.22	16,562	35.62	17595	37.84	
Western Europe	938	2.02	12,743	27.41	13,681	29.42	
Israel	63	0.14	87	1.87	933	2.01	
	Mean	SD	Mean	SD	Mean	SD	
**age**	73.75	10.25	70.19	9.23	70.40	9.33	<0.001

SD, standard deviation.

### 3.2 The prediction of suicidal ideation

The XGBoost model demonstrated robust predictive ability on the test data for predicting suicidal ideation among middle-aged and aged individuals. The XGBoost model achieved an AUC score of 0.80, accuracy of 0.77, sensitivity of 0.69, specificity of 0.77, PPV of 0.16, NPV of 0.98, and F1 score of 0.25. The best parameters were a learning_rate of 0.05 and a maximum depth of 3,200 estimators. Details regarding the importance of early-life and late-life predictors can be found in **Appendix C**. Additionally, we evaluated the predictive ability of the XGBoost model only using early-life predictors, with an AUC score of 0.64, an accuracy of 0.65, a sensitivity of 0.56, a specificity of 0.65, a PPV of 0.09, an NPV of 0.96, and an F1 score of 0.15, which are somewhat lower than those using early-life and late-life predictors together.

### 3.3 Variable importance

**Table 2** presents the importance and ranking of 32 early-life circumstances from six domains in the XGBoost prediction model. In the domain of childhood socioeconomic status, the father education level had the highest importance, suggesting the important role of father education level in children’s suicidal ideation in old age. However, a family’s financial situation during childhood had no obvious effect. It concludes that some socioeconomic circumstances, including parental education and breadwinner occupation, are crucial and others, such as family financial difficulty, may not be direct predictors of suicidal ideation. In the domain of childhood health and healthcare, self-reported health status and access to healthcare resources, such as receiving vaccinations and regularly visiting a dentist, were the strongest predictors, emphasizing the critical role of early health intervention and preventive healthcare in mitigating suicidal ideation in late life. In the domain of childhood war, early exposure to war World War I and World War II had a lower importance in predicting suicidal ideation in old age than most predictors from the domain of childhood health and healthcare. In the domain of childhood relationship, childhood friendship had the most critical effect in prediction, including whether having a group of friends and whether feeing lonely. Living with father during childhood was also a significant predictor. In the domain of childhood residence conditions, the importance of having a cold running water supply ranked first, indicating the importance of basic amenities in affecting children’s future mental health. In the domain of childhood cognition, academic performance in mathematics and language ranked 12 and 10, respectively, suggesting that early cognitive development can influence late suicidal ideation.

**Table 2:** The importance of early-life circumstances in suicidal ideation prediction.

Domains	Predictors	Importance	Ranking of importance
Childhood socioeconomic status	mother_education	0.008150	13
	father_education	0.010683	6
	occupation_breadwinner	0.007983	14
	family_finance	0.000000	29
	living_arrangement	0.009240	8
	religion_importance	0.008660	11
	books	0.005770	27
Childhood health and healthcare	health_before_15	0.012695	3
	missed_school	0.007901	15
	confined_to_bed	0.000000	29
	childhood_diseases	0.007416	18
	childhood_illnesses	0.006424	24
	in_hospital	0.006444	23
	vaccinations	0.015134	2
	dentist_visit	0.009406	7
Childhood war	World_War_I	0.000000	29
	World_War_II	0.006270	26
Childhood relationship	physical_harm	0.007103	20
	lonely_for_friends	0.010788	4
	group_of_friends	0.017511	1
	lived_mother	0.007743	17
	lived_father	0.009224	9
	drank_heavily	0.000000	29
Childhood residence conditions	number_of_rooms	0.006332	25
	number_of_people	0.006485	22
	cold_running_water	0.010745	5
	hot_running_water	0.007324	19
	bath	0.006496	21
	toilet	0.005723	28
	heating	0.007755	16
Childhood cognition	math_performance	0.008151	12
	language_performance	0.008695	10

We further utilized the XGBoost model to identify the ten most crucial early-life circumstances contributing to suicidal ideation among middle-aged (50–59) and older individuals (60+), as detailed in **Table 3**. There was heterogeneity between the two age groups. For instance, the mother education factor was more predictive for middle-aged individuals than for older adults. The predictive performance of the model by age groups is shown in **Appendix E**.

**Table 3:** Top 10 early-life circumstances for middle-aged and aged individuals.

Ranking of importance	Middle-aged individuals (50–59)		Older adults (60+)	
	Predictors	Importance	Predictors	Importance
1	mother_education	0.045515	group_of_friends	0.020894
2	lonely_for_friends	0.033787	living_arrangement	0.012020
3	childhood_diseases	0.028626	father_education	0.011299
4	math_performance	0.026914	health_before_15	0.010497
5	health_before_15	0.025918	hot_running_water	0.010496
6	number_of_rooms	0.025255	vaccinations	0.010413
7	hot_running_water	0.022624	lonely_for_friends	0.010021
8	number_of_people	0.021400	missed_school	0.009966
9	father_education	0.021156	number_of_rooms	0.009016
10	physical_harm	0.019877	lived_father	0.008852

**Figure 2:**
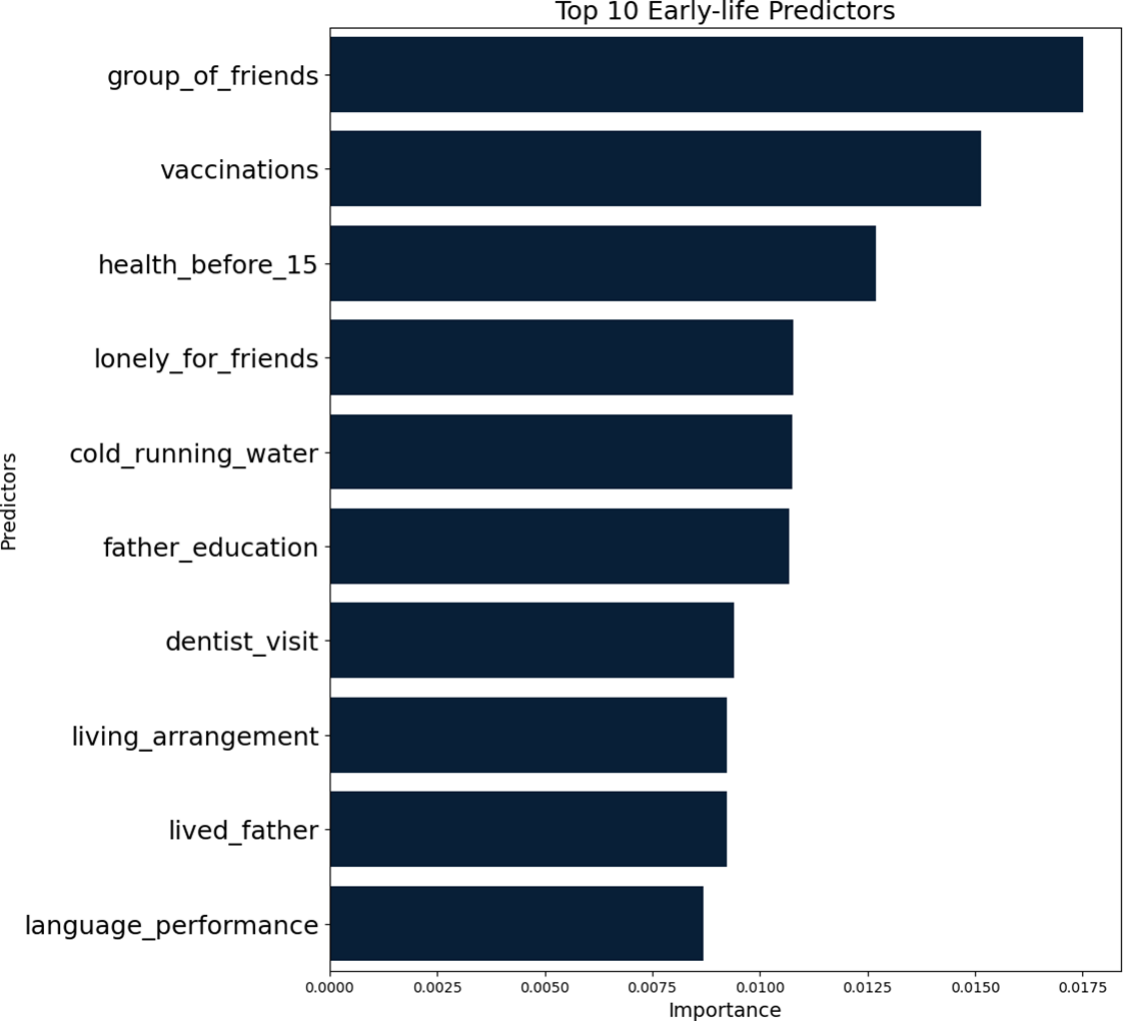
Importance of top 10 early-life circumstances predictors for suicidal Ideation.

**Figure 2** shows the importance of the top 10 early-life predictors in suicidal ideation. The majority of the top 10 predictors were from the domains of childhood relationship (having a group of friends, feeling lonely for friends, and living with father), childhood socioeconomic status (father education and living arrangement), and childhood health and healthcare (vaccination and regularly visiting the dentist). This highlights that predictors during childhood are strongly linked to suicidal ideation in late life.

## 4 Discussion

This study applied the XGBoost model to evaluate the importance of 32 early-life circumstances in predicting suicidal ideation in middle-aged and aged European and Israeli populations using cross-national data. Seventy percent of the study data was used to train the prediction model and 30% was used to assess the trained model. Scale_pos_weight of XGBoost was used to handle the class imbalance problem and 10-fold cross-validation was used to deal with overfitting problem. The performance indicators show the good predictive ability of the XGBoost model, with an AUC of 0.80 and accuracy of 0.77. The results demonstrate that the top predictors are mainly in the domains of childhood relationship, childhood socioeconomic status, childhood health and healthcare, and, especially, whether having a group of friends, which have the most critical influence on suicidal ideation in old age.

To the best of our knowledge, this study is the first to shed light on the relative importance of 32 early-life circumstances from six different domains, leveraging the advantages of the XGBoost predictive model. Some findings align with previous studies emphasizing the importance of childhood socioeconomic status, childhood health and healthcare, childhood resident conditions, and early-life war exposure in predicting suicidal ideation among older adults (8, 11, 15, 37). This study also provides new findings, such as the high importance of childhood friendship and childhood cognition in predicting late suicidal ideation, which lacked sufficient attention in previous studies. Related studies attempted to explore the associations between early-life peer relationships, childhood cognitive function, and mental health in old age (45, 46), but there are still few studies exploring the influence of these childhood factors on late-life suicidal ideation. This study is beneficial in providing new evidence for the targeted and precise intervention to mitigate some lasting effects of early-life circumstances in later suicidal ideation. Additionally, this study emphasized the significance of exploring the causes of suicidal ideation from the life-course perspective.

The top 10 factors among 32 early-life circumstances may inform suicide prevention efforts. These top factors belong to five domains: childhood socioeconomic status, childhood health and healthcare, childhood relationship, childhood residence conditions, and childhood cognition. First, in the domain of childhood relationship, having a group of friends is associated with better social support and mental health. Empirical studies demonstrated that a lack of such a network can lead to loneliness and social barriers, which are known risk factors for suicidal ideation in adulthood (6, 47). This study identified having a group of friends as the most crucial early-life factor for predicting suicidal ideation, aligning with these studies. Therefore, encouraging children to build friendships is crucial in reducing the risk of suicidal thoughts later in life. Feeling lonely is also a critical factor that can lead to persistent mental health problems, including suicidal ideation. Evidence revealed the association between childhood loneliness and a long-term disruption in mental health that extends into adulthood (48). Addressing loneliness in children and adolescents is vital in preventing suicidal behaviors in adulthood. Living with a father provides stable emotional support and healthy socialization patterns. It was demonstrated that a fatherless childhood is associated with higher risks of mental health (49). This suggests that maintaining a supportive family environment is crucial for suicide prevention.

Second, in the domain of childhood health and healthcare, vaccinations had a long-term impact on health. A study found that vaccinations before the age of 15 have positive associations with cognitive and educational outcomes (50), which may decrease the risks of mental health. Thus, improving access to vaccinations is beneficial for indirectly decreasing suicide risk. Early health conditions persistently impact health outcomes in late life. Poor health in childhood is associated with higher odds of work-limiting disability and chronic health conditions (51), which may increase the suicide risk. In terms of suicide prevention, addressing early health concerns allows for timely interventions that may decrease the risk of later suicidal ideation. Regular dental visits indicate a family’s exposure to preventive care. Evidence shows that regular dental visits during childhood positively impacts the quality of life in old age (52). Improving the availability of preventive care for children may lower the risk of suicide in old age.

Third, in the domain of childhood residence conditions, residential conditions such as access to cold running water affect children’s mental and physical health. Research has shown that poorer residential conditions are linked to adult mortality (53). Therefore, improving early residential conditions is an effective strategy for reducing long-term health issues, ultimately aiding in suicide prevention. Fourth, in the domain of childhood socioeconomic status, father education often correlates with the family’s socioeconomic status, in which higher education levels can decrease the risks of depression and other mental health problems (54). Focusing on individuals from disadvantaged childhood socioeconomic backgrounds may reduce their risk of developing suicidal ideation. Difficult living arrangements, including living in an orphanage and being fostered by another family, may cause emotional and behavioral problems, increasing long-term psychological stress. Evidence has proven the associations between living in an orphanage and cognitive disease in late life (55). Early intervention measures of paying more attention to children with difficult living arrangements can reduce adult mental health issues. Finally, in the domain of childhood cognition, early language performance has an impact on late-life health. A study has shown that low linguistic ability in early life is a strong predictor of poor cognitive function in late life (56). Enhancing language performance may improve social interactions and psychological adjustments, thus reducing suicide risk.

The study has strengths in four aspects. First, it used a large set of early-life circumstances from six domains, providing a comprehensive analysis of how different early-life circumstances contribute to the risk of suicidal ideation in old age. Second, this study used the cross-national data that included the majority of European countries, rather than data of a single country, enhancing the generalizability of the identified early-life risks. Finally, this study considered the heterogeneity of the importance of early-life circumstances for late-life ideation among middle-aged individuals and older adults, which is crucial for designing targeted interventions based on age.

There are several limitations to this study. First, the information about suicidal ideation was self-reported by participants, and early-life circumstances were based on the participants’ recall, which may introduce information bias. Second, there was a survival bias in this study as individuals with adverse early-life circumstances may have died earlier and therefore were not included in the study. Third, this study identified the association between early-life circumstance and late-life suicide rather than examining the causality. Additionally, the XGBoost predictive model was developed using SHARE data that primarily includes high-income countries. It is uncertain whether the model will be robust when applied to data from middle- and low-income countries.

## 5 Conclusion

In this study, the findings illustrate that early-life circumstances play a crucial role in predicting suicidal ideation in late life, especially the predictors in the domains of childhood relationship, childhood socioeconomic status, and childhood health and healthcare. The predictors identified in this study were in line with previous studies, reaffirming the long-term effect of early-life circumstances on suicidal ideation. In addition, this study highlights the promise of machine learning models in identifying the risk for suicidal ideations. Based on our findings, several preventive measures can be helpful to lower the risk of suicidal ideation in late life. On one hand, early-life stage interventions are crucial. For example, encouraging children to build friendships, improving access to preventive care such as vaccinations, improving early health conditions, and improving residential conditions, may contribute to lowering the risk of suicidal ideation in late life. On the other hand, the identified key early-life circumstances, such as the top ten circumstances, may be used to screen older populations for further diagnoses and care. However, there is the heterogeneity of the importance of early-life circumstances for late-life ideation between middle-aged individuals and older adults. The heterogeneity is crucial for designing age-specific interventions when screening susceptible populations.

## Data Availability

Publicly available datasets were analyzed in this study. This data can be found here: SHARE (the Survey of Health, Ageing and Retirement in Europe), https://share-eric.eu/.
