# Supplementary material for: Do early-life circumstances predict late-life suicidal ideation? Evidence from SHARE data using machine learning
Source: Front Psychiatry. 2024 Nov 14;15:1426876. doi: 10.3389/fpsyt.2024.1426876 (PMC11602597; doi:10.3389/fpsyt.2024.1426876)
Supplement: Supplementary file 1 [file DataSheet1.docx]

# Appendix A

Detailed description of these predictors of early-life circumstances.

| Domains | Predictors | Meaning of predictors |
| --- | --- | --- |
| Childhood socioeconomic status | mother__education | The education level of the respondent’s mother  0. None  1. Primary education  2. Lower secondary education  3. Upper secondary education  4. Post-secondary non tertiary education  5. First stage of tertiary education  6. Second stage of tertiary education |
|  | father_education | The education level of the respondent’s father  0. None  1. Primary education  2. Lower secondary education  3. Upper secondary education  4. Post-secondary non tertiary education  5. First stage of tertiary education  6. Second stage of tertiary education |
|  | occupation_breadwinner | The occupation of main breadwinner when respondent was 10 years  1. Legislator, senior official or manage  2. Professional  3. Technician or associate professional  4. Clerk  5. Service, shop or market sales worker  6. Skilled agricultural or fishery worke  7. Craft or related trades worker  8. Plant/machine operator or assembler  9. Elementary occupation  10. Armed forces  11. Spontaneous only: there was no main |
|  | family_finance | Whether respondent experienced financial hardship before age 16  0. No  1. Yes |
|  | living_arrangement | Whether respondent experienced difficult living arrangement before age 16  0. No  1. Yes |
|  | religion_importance | The importance of religion at home when respondent grew up  1. Very important  2. Somewhat important  3. Not very important  4. Not at all important |
|  | books | The number of books in household when respondent was 10 years  1. None or very few (0-10 books)  2. 1 bookshelf (11-25 books)  3. 1 bookcase (26-100 books)  4. 2 bookcases (101-200 books)  5.3+ bookcases (more than 200 books) |
| Childhood health and healthcare | health_before_15 | Respondent’s self-reported health status during childhood  1. Excellent  2. Very good  3. Good  4. Fair  5. Poor  6. Health varied a great deal |
|  | missed_school | Whether respondent missed school for 1+ months due to health before age 16  0. No  1. Yes |
|  | confined_to_bed | Whether respondent was confined to bed or home for 1 month or longer  5. No  1. Yes |
|  | childhood_diseases | Whether respondent had any of the diseases before age 16 (Infectious disease, Polio, Asthma, Respiratory problems other than asthma, Allergies, Severe diarrhoea, Meningitis/encephalitis, Chronic ear problems, Speech impairment, Difficulty seeing even with eyeglasses)  0. No  1. Yes |
|  | childhood_illnesses | Whether respondent had any of the illness before age 16 (Severe headaches or migraines, Epilepsy, fits or seizures, Emotional, nervous, or psychiatric problem, Broken bones, fractures, Appendicitis, Childhood diabetes or high blood sugar, Heart trouble, Leukaemia or lymphoma, Cancer or malignant tumour (excluding minor skin cancers), Rickets, osteomalacia, rachitis)  0. No  1. Yes |
|  | in_hospital | During childhood, because of a health condition, whether respondent ever was in hospital for one month or more  5. No  1. Yes |
|  | vaccinations | Whether respondent had vaccination in childhood  0. No  1. Yes |
|  | dentist_visit | Whether respondent started going regularly to the dentist before age 16  5. No  1. Yes |
| Childhood war | World_War_I | Whether respondent was born between 1914 and 1918  0. No  1.Yes |
|  | World_War_II | Whether respondent was born between 1939 and 1945  0. No  1. Yes |
| Childhood relationship | physical_harm | Whether respondent was physically harmed by anybody else in any way  1. Often  2. Sometimes  3. Rarely  4. Nerve |
|  | lonely_for_friends | How often respondent felt lonely for friends in childhood  1. Often  2. Sometimes  3. Rarely  4. Nerve |
|  | group_of_friends | Between ages 6-16, how often respondent had a group of friends that felt comfortable spending time with  1. Often  2. Sometimes  3. Rarely  4. Nerve |
|  | lived_mother | Whether respondent’s mother lived when respondent was 10 years  0. No  1. Yes |
|  | lived_father | Whether respondent’s father lived with when respondent was 10 years  0. No  1. Yes |
|  | drank_heavily | Whether respondent’s parents or guardians drank heavily or had mental health problem  0. No  1. Yes |
| Childhood residence conditions | number_of_rooms | The number of rooms in accommodation when respondent was 10 years |
|  | number_of_people | The number of people living in household when respondent was 10 years |
|  | cold_running_water | Whether there had cold running water supply in accommodation when respondent was 10 years  0. No  1. Yes |
|  | hot_running_water | Whether there had hot running water supply in accommodation when respondent was 10 years  0. No  1. Yes |
|  | bath | Whether there had fixed bath in accommodation when respondent was 10 years  0. No  1. Yes |
|  | toilet | Whether there had inside toilet in accommodation when respondent was 10 years  0. No  1. Yes |
|  | heating | Whether there had central heating in accommodation when respondent was 10 years  0. No  1. Yes |
| Childhood cognition | math_performance | Respondent’s relative performance of mathematics when 10  1. Much better  2. Better  3. About the same  4. Worse  5. Much worse |
|  | language_performance | Respondent’s relative performance of language when 10  1. Much better  2. Better  3. About the same  4. Worse  5. Much worse |

# Appendix B

Detailed description of late-life predictors

| Domains | Predictors | Meaning of predictors |
| --- | --- | --- |
| Demographic predictors | gender | Respondent’s gender  1. Man  2. Woman |
|  | age | Respondent’s age at interview |
|  | marrital_status | Respondent’s marital status  1. Married  3. Registered partnership  4. Separated  5. Divorced  7. Widowed  8. Never married |
|  | education | Respondent’s education level  1. Less than upper secondary education  2. Upper secondary and vocational training  3. Tertiary education |
|  | rural_urban | Whether respondent lives in rural or urban area  0. Urban  1. Rural |
|  | living_in_nursing_home | Whether respondent lives in nursing homes  0. No  1. Yes |
|  | region | Region where respondent lives  1. Northern European countries (Denmark, Sweden, Finland)  2. Southern European countries (Spain, Italy, Greece, Cyprus, Malta, Portugal)  3. Eastern European countries (Slovenia, Estonia, Lithuania, Bulgaria, Latvia, Romania, Poland, Hungary, Slovakia, Czech Republic, Croatia)  4. Western European countries (Netherlands, France, Belgium, Ireland, Luxembourg, Austria, Germany, Switzerland)  5. Israel |
| Health and healthcare predictors | ADL | Whether respondent experiences difficulties with any of the five ADL activities (bath, dress, eat, getting in/out of bed, walk across a room) |
|  | IADL | Whether respondent experiences difficulties with any of the seven IADL activities (using the phone, managing money, taking medications, shopping for groceries, preparing hot meals, using a map, and doing housework) |
|  | mobile | Whether respondent experiences difficulties with any of the seven activities (walking across a room, climbing several flights of stairs activities, getting up from a chair, stooping, lifting 10 pounds, extending arms up, and picking up a small coin activities ) |
|  | companion | How much time the respondent feels they lack companionship    1. Hardly ever or never  2. Some of the time and  3. Often |
|  | lefout | How much time the respondent feels left out    1. Hardly ever or never  2. Some of the time and  3. Often |
|  | isolate | How much time the respondent feels isolated from others  1. Hardly ever or never  2. Some of the time and  3. Often |
|  | loneliness | Respondent’s 3-item loneliness (feels they lack companionship, feels left out, feels isolated from others) summary mean score |
|  | self_health | Respondent’s self-report of health  1. Excellent  2. Very good  3. Good  4. Fair  5. Poor |
|  | cognition | Respondent’s cognition orient (the summary measure for the orientation to date, month, year and day of the week) |
|  | smoke | Whether respondent smokes now  0. No  1. Yes |
|  | drink | Whether respondent drinks alcohol in the last 7 days  0. No  1. Yes |
| Economic predictors | pension | Whether respondent receives public pensions  0. No  1. Yes |
|  | income | Respondent’s household total income |
| Social network predictors | weekly_contact | Whether respondent has any weekly contact with children in person/ phone/email  0. No  1. Yes |
|  | frequency_contact | Respondent’s frequency of contacting with children in person/ phone/email  1. Daily  2. Several times a week  3. About once a week  4. About every two weeks  5. About once a month  6. Less than once a month  7. Never |
|  | social_activity | Whether the respondent has any yearly social activities  0. No  1. Yes |

# Appendix C

Importance of early-life and late-life predictors for suicidal ideation.


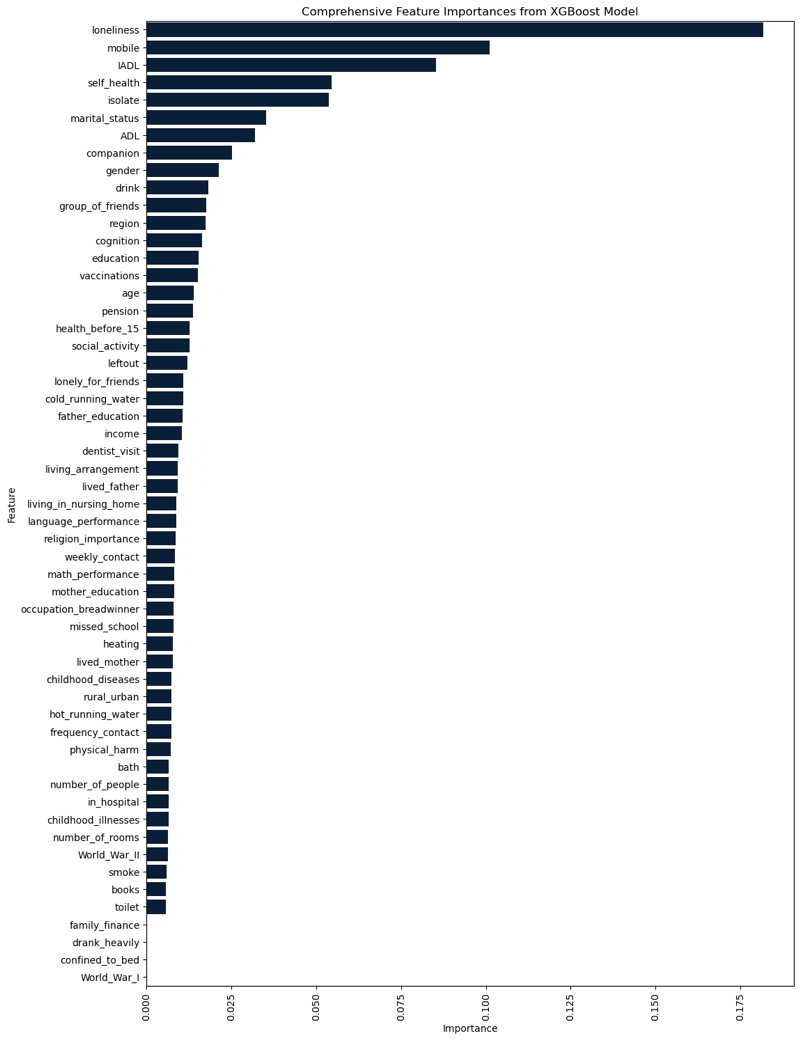


# Appendix D

Correlation matrix as a heatmap for all predictors


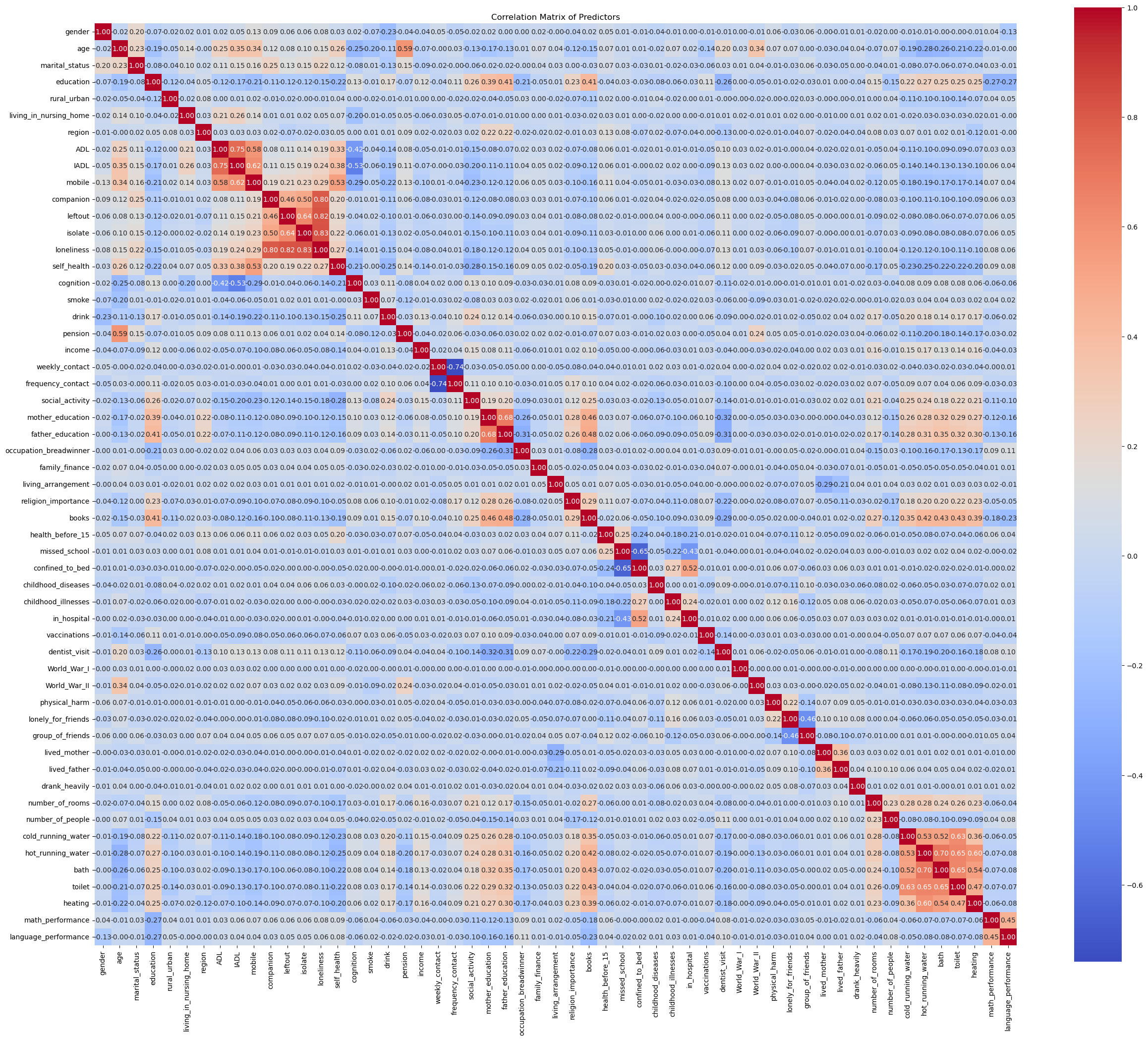


# Appendix E

Model predictive performance by age groups.

For the overall sample, the model achieved an AUC score of 0.80, an accuracy of 0.77, a sensitivity of 0.69, a specificity of 0.77, a positive predictive value (PPV) of 0.16, and a negative predictive value (NPV) of 0.98. For samples of middle-aged individuals (50-59), the model’s performance was AUC = 0.80, accuracy = 0.79, sensitivity = 0.68, specificity = 0.80, PPV = 0.12, and NPV = 0.98. For samples of aged individuals (60+), the performance was AUC = 0.81, accuracy = 0.77, sensitivity = 0.72, specificity = 0.77, PPV = 0.18, and NPV = 0.98. These metrics suggest that the model performed similarly across age groups, with slight variations in several metrics.
